# Case Report: Use of extracorporeal support to treat a fulminant *Mycobacterium bovis* infection and complex broncho-oesophageal fistula in a child

**DOI:** 10.3389/fped.2025.1559240

**Published:** 2025-09-05

**Authors:** Hussein Hussein, Richard Paget, Louis Grandjean, Alasdair Bamford, Ashwin Pandey, Katy Fidler, Claire Snowden, Arun Beeman, Pooja Shetty, Nagarajan Muthialu

**Affiliations:** ^1^ Departments of Paediatric Cardiac Intensive Care Unit, Great Ormond Street Hospital NHS Foundation Trust, London, United Kingdom; ^2^ Departments of Infectious Diseases, Great Ormond Street Hospital NHS Foundation Trust, London, United Kingdom; ^3^ Departments of General Paediatrics, University Hospitals Sussex NHS Foundation Trust, Brighton, United Kingdom; ^4^ Departments of Paediatric Cardiothoracic Surgery, Great Ormond Street Hospital NHS Foundation Trust, London, United Kingdom

**Keywords:** ECMO, mycobacterium, broncho-oesophageal fistula, pneumonectomy, oesophageal surgery

## Abstract

Tuberculosis (TB), caused by *Mycobacterium tuberculosis* remains a significant public health issue. Human TB caused by *Mycobacterium bovis (M. bovis) is rare* accounting for less than 1% of TB cases in UK annually. Tuberculosis secondary to immunomodulating agents is well described. We present a case of airway and pulmonary TB caused by *M. bovis*, likely due to zoonotic transmission, in an immunocompromised child due to medical management of Crohn's disease. Management required extracorporeal membrane oxygenation for complex surgical interventions on airway and oesophagus.

## Introduction

Tuberculosis (TB) caused by *Mycobacterium bovis* (*M. bovis*) accounts for less than 1% of TB cases annually in the UK and is rare in children (1). Human infection typically occurs through ingestion of unpasteurized milk or inhalation of mycobacteria from infected animals (1, 2). TB-associated bronchoesophageal fistula (BOF) is a rare complication and often fatal, typically resulting from erosion of lymph nodes or bronchial inflammation (2, 3). Immunocompromised state, in particular, tends to predispose to or reactivate tuberculosis, and are often associated with complicated forms including development of erosion, necrosis and/or fistula formation. There is a paucity of literature describing the management of BOF in intrathoracic TB in children (3).

## Case presentation

**Figure 1:**
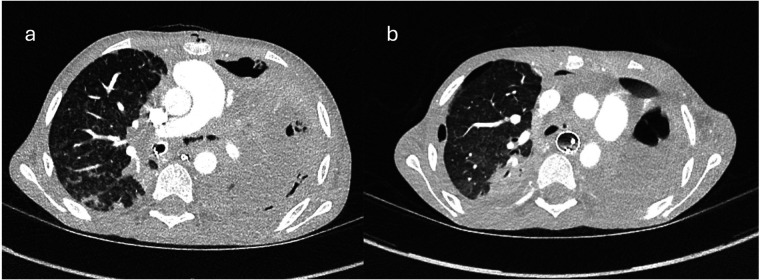
Ctscan images showing **(a)** consolidated left lung, endotracheal tube in right bronchus and irregular left main bronchus; **(b)** free mediastinal air at the level of LMB indicating defect in bronchus.

A 12-year-old child with Crohn's disease, on infliximab for three months, presented with a nine-day history of high-grade fever, anorexia, weight loss and dry cough. Initial computerised tomographic (CT) scan of chest revealed extensive consolidation of left lung (Figure 1), with a large pleural effusion and hilar and mediastinal lymphadenopathy. He was intubated due to respiratory deterioration and subsequent bronchoalveolar lavage revealed infection with *M. bovis* on polymerase chain reaction (PCR). Despite starting appropriate anti-TB medications (Rifampicin, Isoniazid, Moxifloxacin and Ethambutol) there was no clinical improvement. While there was no evidence of any fistula on the CT images, the appearance of left main bronchus was abnormal at this stage.

**Figure 2:**
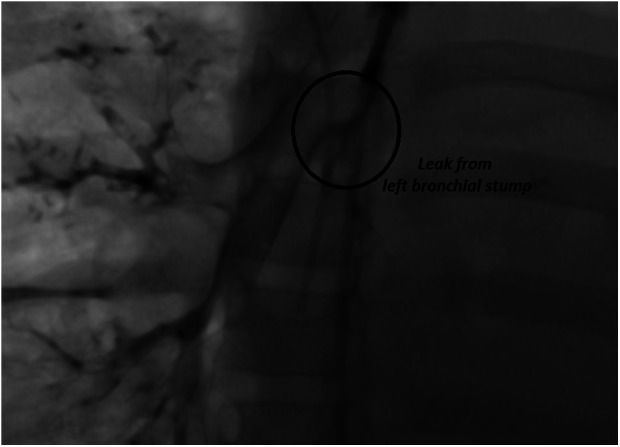
Bronchographic image showing leak of contrast into mediastinum from left main bronchus and contrast within oesophagus confirming BOF.

**Figure 3:**
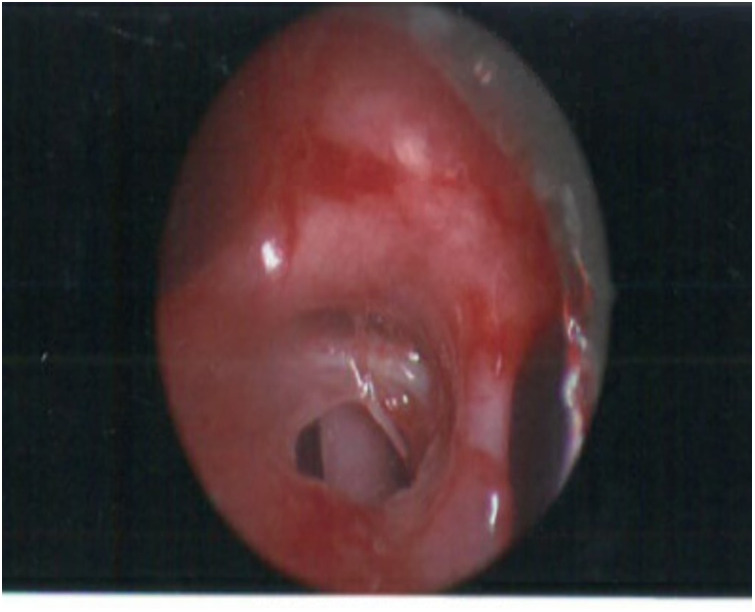
Bronchoscopic image showing a posterior defect in left main bronchus withh oesophagus with nasogastric tube.

Continued clinical deterioration needed initial bronchoscopy (Figures 2, 3), which confirmed broncho-oesophageal fistula involving left main bronchus. Subsequently, oesophagoscopy was also performed to confirm the presence of fistula. The clinical status at this stage, in the presence of fistula between left main bronchus and oesophagus, warranted use of femoro-femoral veno-venous extracorporeal membrane oxygenation (ECMO), as a means for gas exchange for ongoing air leak and respiratory failure. Following this, surgery was undertaken under cardiopulmonary bypass, wherein autologous pedicled pericardial patch was used to reconstruct the bronchus with direct repair for oesophageal defect. The initial course after surgery was uneventful, but the course was complicated further by recurrence of defect with dehiscence of the patch resulting in recurrent BOF.

At repeat surgery, the most part of left lung was necrotic. Pneumonectomy was performed and the bronchial stump was reconstructed using additional pericardial patch as reinforcement. The oesophageal defect was repaired directly but supported with intraluminal stent. He required veno-arterial (VA) ECMO support in the immediate postoperative period due to vasoplegic shock, with severe systemic inflammatory reaction, which was weaned off after 4 days.

Management beyond this continued to be challenging, with communicating pleural cavities and spilling of pleural collections into mediastinum. This contributed to post-pneumonectomy physiology and management involved careful chest drain management with intermittent drainage.

He was extubated after 5 months with nocturnal low flow oxygen requirement. The oesophageal stent was removed 2 months later and oral feeds commenced after swallowing assessment.

The follow-up care involved several specialized teams with contributions from Infectious diseases team (for antituberculous therapy), surgical team (oesophageal recovery), respiratory team (respiratory support) and gastroenterology (management of Crohn's disease and relevant immunosuppressive therapy). He remains asymptomatic at 16 months after surgery.

## Clinical discussion

TB BOF is an extremely rare condition with only a small number of cases reported in literature. There is a single large case series reported by Goussard et al. who reported a total of 20 children diagnosed with TB-associated BOF between 1999 and 2019, with a 75% survival (3, 4). Immunocompromised children are at a higher risk of severe TB and its complications, including fistula formation. Rana et al. reported a case of tuberculous BOF in steroid-responsive nephrotic syndrome. All these reports are on cases with BOF due to *Mycobacterium tuberculosis* (5).

We report a case of fulminant *M. bovis* infection in a 12-year-old child in an immunocompromised state secondary to Crohn's disease. Management of this life-threatening case required ECMO support and multiple complex surgical interventions, demonstrating the potential severity of this infection in paediatric patients.

Diagnosing *M. bovis* infection in a paediatric patient presents significant challenges due to its rarity and non-specific clinical presentation. The initial presentation was more generic with respiratory and gastrointestinal symptoms but the diagnosis involved the use of bronchoalveolar lavage and accurate PCR testing in identifying *M. bovis*, highlighting the importance of advanced diagnostic tools in detecting rare pathogens. Additionally, the chest CT provided critical insights into the extent of pulmonary involvement, revealing pulmonary and airway involvement. Bronchoscopy and oesophagoscopy confirmed large left BOF.

BOF is a rare and severe complication of tuberculosis, often resulting from either the erosion of tuberculous lymph nodes into adjacent structures or direct bronchial necrosis secondary to fulminant infection and inflammation of bronchial wall (6–8). Predilection of LMB involvement is selective as seen in other studies in the literature (6, 7).

The management of *M. bovis* infection required an aggressive and multifaceted approach. At the initial stage itself, despite the initiation of appropriate anti-tuberculosis therapy, further deterioration of clinical status necessitated use of ECMO for hemodynamic stability. There was a large fistula between left bronchus and oesophagus, impacting stable ventilation due to sustained air leak. Additional pulmonary changes with consolidation and evolving pleural effusion complicated the respiratory status further. In view of this, ECMO was initially instituted to stabilise the respiratory status. This was achieved in the form of veno-venous ECMO between femoral vein to other femoral vein.

Surgery involved initial corrective procedure—wherein autologous pedicled pericardial patch (as a vascularised patch) was used to reconstruct the defect in left main bronchus. The defect in oesophagus was repaired directly. Extensive mediastinal debridement including removal of necrotic lymph nodes was performed at this stage as part of surgery.

In spite of corrective surgery, the patch dehisced, partly due to continued inflammation and extensive infective necrosis in the tissues around the area of repair, resulting in further recurrent fistula. With extensive necrosis in the left lung at this stage, the decision was to proceed with pneumonectomy, there by the diseased and necrotic lung was removed. The bronchial stump was repaired, with additional pericardial patch for reinforcement. The oesophageal defect was repaired again directly, but at this stage, an intraluminal stent was used to further support the repaired oesophageal repair.

He continued to need ECMO in the postoperative period, due to ongoing vasoplegic shock. This was instituted as veno-arterial ECMO placed centrally with cannula between aorta and right atrium. Once recovered from the severe systemic inflammatory reaction and shock, ECMO was weaned off after 4 days.

The surgical challenges were exacerbated by the extensive inflammation and tissue friability. The resultant ongoing necrosis needed repeat surgery in view of recurrent fistula formation, resulting also in pneumonectomy.

Management of oesophagus involved difficulties with choice and sizing of stent for recurrent fistula to avoid both erosion and dislodgement. A larger stent would potentially involve risk for tracheal compression (4).

Goussard et al. demonstrated that management of BOF can be by spontaneous closure, surgery via thoracotomy, use of fibrin glue and oesophageal stents (4, 8).

The successful management of our case was heavily reliant on a multidisciplinary approach involving infectious disease specialists, thoracic surgeons, general surgeons and intensive care specialists.

## Conclusion

Fulminant *M. bovis* infection with BOF is rare and life-threatening. Effective management requires a combination of anti-TB treatments, surgical interventions, and multidisciplinary care. This case highlights the need for comprehensive treatment strategies including appropriate surgical and non-surgical interventions as well as ECMO when clinically needed.

## Data Availability

The raw data supporting the conclusions of this article will be made available by the authors, without undue reservation.
